# Supplementary material for: Socio-economic and demographic determinants of fertility in six selected Pacific Island Countries: An empirical study
Source: PLoS One. 2021 Sep 22;16(9):e0257570. doi: 10.1371/journal.pone.0257570 (PMC8457485; doi:10.1371/journal.pone.0257570)
Supplement: Attachment — Submitted filename: comment_PONE-D-21-02736.docx [file pone.0257570.s002.docx]

**Determinants of Fertility Rates in Selected Pacific Islands Countries: A Multi-Level Empirical Study**

**OVERALL FEEDBACK:** This study examines an important topic— the determinants of fertility rates in selected pacific island countries. The authors explore a range of different variables- women’s empowerment, health, connectivity, contraceptive prevalence, unmet need for family planning and exposure to coitus mainly influence fertility rates of the Pacific countries, which contributes to the literature on fertility trends of these countries. The paper requires some improvements before it could be considered for publication. First, the introduction is quite long and readers may struggle to understand the research problem the authors are trying to address. It is not clear the research question(s) and the new knowledge the study will contribute. Authors should state the main hypothesis being tested or the research question being addressed. The methods section should be improved by providing more details on the data sources, including data quality and limitations, summarize the socio-economic and cultural context of the five countries which might influence the findings. I recommend authors refer to standard quantitive research reporting standards (JARS-Quant). The authors should also separate the results and discussion sections. Other specific suggestions, which I hope will improve the clarity and contribution of this important work, are itemized by pieces below.

**Abstract**

- Indicate the data sources, sample size, and the main analysis model use.
- To what extent does women’s empowerment, health, contraceptive prevalence and exposure to coitus mainly influence fertility rates in the 6 countries.
- The conclusion should be based on the findings

**Introduction**

- The introduction is rather too long with a reproduction of various statistics already available in reports. It is therefore difficult to understand what research gap the authors are studying and the new knowledge they are contributing. The way the introduction is currently written would not fit most journal standards. The authors should summarise the introduction into 2-3 succinct paragraphs.
- It is unclear the main objective of the study. The authors say their study aims to review the current rate of knowledge on the fertility rate in the Pacific but go further to conduct a multi-level empirical analysis on determinants of fertility. It is, therefore, not clear whether the authors want to conduct a desk review or do an analysis on the determinants of fertility.
- Outlining a conceptual model would be useful in showing the expected relationship between factors being examined. The authors should also highlight key hypotheses to be tested in the study.
- The socio-economic and demographic context of the 6 countries (Line 164-364) can be summarised into a table or a graph to limit the number of paragraphs
- Ln 141- This research is important because such studies are limited and out-of-date-which are these out-date literature authors are talking about?
- Line 142, the authors wrote “Thus, providing empirical support to understanding the causes of fertility can help countries rejuvenate their population, reduce the high dependency of labour force and manage their youth bulge. It is not clear what the author refers to when they say ‘rejuvenate their population’. What is the main issue here, is it high/low population growth? It appears the challenge being addressed is high pop growth but later on the authors mention the problem of one-child families.
- Line 160-163 is not necessary for journal articles
- Line 150- the justification of using both macro and household-level data to model the determinants of fertility is not adequate.
- Line 365-585 “survey of literature” This section can be summarized into one/two paragraph(s) and merged with the introduction section.

**Methods**

- Need to describe the study settings- provide the demographic, cultural, social, and economic context of the six countries included in the analysis. A table will do
- Data source- provide more nuances on the data sources, data quality, and limitations.
- Provide a justification why the two datasets are used
- What guided the type of variables included in the analysis
- Indicate how the dependent and independent variables included in the analysis were measured
- State why doing both micro and macro analysis is necessary. The two levels of analysis can not be compared given that some of the variables used do not cut across both. I would suggest authors just stick with one type of analysis.
- Line 595 the authors wrote “ a cross-sectional random sample of household-level analysis is presented, followed by a more general economy-wide panel data based aggregated estimates” what was the sampling frame, how was the sample size derived?
- Line 600 “A response rate of 97% was maintained by using a standard questionnaire that enquired details of fertility rates, issues and determinants” It is unclear what the subject matter here is, that is, who was interviewed, their inclusion and exclusion criteria, what questions were asked, how were the participants recruited e.t.c.
- Line 602 “All standards of research ethics were followed in obtaining, using and storing the relevant household data’ the authors should indicate how the ethics were ensured, i.e was IRB approval granted and by which institution.
- There is a need to conduct the unadjusted and adjusted analysis to estimate the gross and net effect of each of the independent variables.
- Include the assumptions made based on the model used

**Findings**

- Would suggest redoing this section and consider having a separate section for the results and discussion. Describe the results for micro and macro analysis separately. The discussion should focus more on the interpretation of the results, comparison with other findings, generalizability, and implications of the findings

**Conclusion**

- It is important to recognize that fertility and economic development are causally related
- It is unclear what the authors meant in line 700. The authors wrote “... family formation and the education and employment systems should not drag women longer to prove to themselves”
- Some of the conclusions are not supported by the findings. For example, “…the society must be educated of the moral and social obligations of women, including their expectations”
