# Supplementary material for: Socio-economic and demographic determinants of fertility in six selected Pacific Island Countries: An empirical study
Source: PLoS One. 2021 Sep 22;16(9):e0257570. doi: 10.1371/journal.pone.0257570 (PMC8457485; doi:10.1371/journal.pone.0257570)
Supplement: Attachment — Submitted filename: Response to Reviewers.docx [file pone.0257570.s003.docx]

“Socio-Economic and Demographic determinants of Fertility in Six Selected Pacific Islands Countries: An Empirical Study” ( PONE-D-21-02736)

First Revision : ( 30^th^ August ) Reviewers’ comments and Authors’ responses:

|  | **Reviewer 1 – Major comments** | Author response |
| --- | --- | --- |
|  | Overall feedback |  |
|  | First, the introduction is quite long and readers may struggle to understand the research problem the authors are trying to address. It is not clear the research question(s) and the new knowledge the study will contribute. Authors should state the main hypothesis being tested or the research question being addressed. The methods section should be improved by providing more details on the data sources, including data quality and limitations, summarize the socio-economic and cultural context of the five countries which might influence the findings. I recommend authors refer to standard quantitive research reporting standards (JARS-Quant). The authors should also separate the results and discussion sections. Other specific suggestions, which I hope will improve the clarity and contribution of this important work, are itemized by pieces below. | We really appreciate the reviewers for their valuable comments. We have now embedded the necessary changes to our paper and the changes have been identified below.  Introduction is now conscise and it clearly addresses the research questions and new knowledge that this study contributes. The hypothesis being tested is explained in introduction, with a more details being included under methods sections, along with the reasons variable & model selection. More details on the data sources, including data quality and limitations has been included in methods section, as well as a tabulated version of socio-economic and demographic context of the six countries. The results and discussion section are now easily distinguishable. |
| 1. | **Abstract.**  -Indicate the data sources, sample size, and the main analysis model use.  - To what extent does women’s empowerment, health, contraceptive prevalence and exposure to coitus mainly influence fertility rates in the 6 countries.  -The conclusion should be based on the findings | Data sources, sample size, and the main analysis model used in the analysis has been indicated  The sign for the main variables, as well as their significance level has been stated.  This comment has been now addressed as part of the conclusions. |
| 2. | **Introduction.**  -The introduction is rather too long with a reproduction of various statistics already available in reports. It is therefore difficult to understand what research gap the authors are studying and the new knowledge they are contributing. The way the introduction is currently written would not fit most journal standards. The authors should summarise the introduction into 2-3 succinct paragraphs.  - It is unclear the main objective of the study. The authors say their study aims to review the current rate of knowledge on the fertility rate in the Pacific but go further to conduct a multi-level empirical analysis on determinants of fertility. It is, therefore, not clear whether the authors want to conduct a desk review or do an analysis on the determinants of fertility.  - Outlining a conceptual model would be useful in showing the expected relationship between factors being examined. The authors should also highlight key hypotheses to be tested in the study.  -The socio-economic and demographic context of the 6 countries (Line 164-364) can be summarised into a table or a graph to limit the number of paragraphs  - Ln 141- This research is important because such studies are limited and out-of-date-which are these out-date literature authors are talking about?  - Line 142, the authors wrote “Thus, providing empirical support to understanding the causes of fertility can help countries rejuvenate their population, reduce the high dependency of labour force and manage their youth bulge. It is not clear what the author refers to when they say ‘rejuvenate their population’. What is the main issue here, is it high/low population growth? It appears the challenge being addressed is high pop growth but later on the authors mention the problem of one-child families.  - Line 160-163 is not necessary for journal articles  - Line 150- the justification of using both macro and household-level data to model the determinants of fertility is not adequate.  - Line 365-585 “survey of literature” This section can be summarized into one/two paragraph(s) and merged with the introduction section. | The entire introduction has been re-written and reproduced statistics have been either dropped or presented as a summary in table.  The objective of the study has now been made clear in the text.  Conceptual framework has been brefly outlined towards the end of introduction, along with the key hypothesis. More detailed version of hypothesis can be found under methods  The 6 country contexts have been summarised into a table  Ln 141 on outdated research has been now identified and stated in text.  This statement has been completely removed  It has been removed  Micro-analysis has been removed, as per the reviewwers suggestion and the justification of macro-level data is provided in introduction and also under methods section.  Survey of literature has now been summarized and merged with introdution |
| 3. | **Methods.**  -Need to describe the study settings- provide the demographic, cultural, social, and economic context of the six countries included in the analysis. A table will do  - Data source- provide more nuances on the data sources, data quality, and limitations.  -Provide a justification why the two datasets are used  **-** What guided the type of variables included in the analysis  - Indicate how the dependent and independent variables included in the analysis were measured  - State why doing both micro and macro analysis is necessary. The two levels of analysis can not be compared given that some of the variables used do not cut across both. I would suggest authors just stick with one type of analysis.  - Line 595 the authors wrote “ a cross-sectional random sample of household-level analysis is presented, followed by a more general economy-wide panel data based aggregated estimates” what was the sampling frame, how was the sample size derived?  - Line 600 “A response rate of 97% was maintained by using a standard questionnaire that enquired details of fertility rates, issues and determinants” It is unclear what the subject matter here is, that is, who was interviewed, their inclusion and exclusion criteria, what questions were asked, how were the participants recruited e.t.c.  - Line 602 “All standards of research ethics were followed in obtaining, using and storing the relevant household data’ the authors should indicate how the ethics were ensured, i.e was IRB approval granted and by which institution.  -There is a need to conduct the unadjusted and adjusted analysis to estimate the gross and net effect of each of the independent variables.  - Include the assumptions made based on the model used | This has been addressed in the form of table  This has been now accounted for under data sources and limitations section  Now only Macro-level dataset is used, along with its justification  This has been now explained under model specification section. Frameworks of Davis & Black(1956) and Bongaarst(1987) guided the variable selection  This has been indicated under data sources and limitations section  Now only Macro-level dataset is used, as per the suggestions from the reviewer.  Household level analysis has been removed from this study, detailes now not needed.  Household level analysis has been removed from this study – we do not need these anymore.  All relevant research ethics were followed for obtaining and using household data. Now since micro analysis are removed,the approval (although we have obtained) is not required.  The net effect of the predictors on the outcome variable is examined by removing the influence of those time-invariant features using FE.  This has been now included under estimation techniques |
| 4. | **Findings**  -Would suggest redoing this section and consider having a separate section for the results and discussion. Describe the results for micro and macro analysis separately. The discussion should focus more on the interpretation of the results, comparison with other findings, generalizability, and implications of the findings | Micro-analysis has been removed and this section has been redone, with appropriate re-estimations and additional statistics being included in the analysis. The results and discussion section are now easily distinguishable. The implication has been stated in brief under findings, but has bene comprehensively covered under conclusion and implications section |
| 5. | **Conclusion**  **-** It is important to recognize that fertility and economic development are causally related  - It is unclear what the authors meant in line 700. The authors wrote “... family formation and the education and employment systems should not drag women longer to prove to themselves”  - Some of the conclusions are not supported by the findings. For example, “…the society must be educated of the moral and social obligations of women, including their expectations” | This has been now explained  This sentence has been removed  This sentence has been removed |
|  | **Reviewer 2** |  |
|  | General Comments |  |
|  | The topic covered in the article could be an interesting read for researchers. The manuscript attempts to explore the nuances of various indicators on fertility in selected six Pacific Islands countries. The manuscript is excessively lengthy and severely lacks coherence in both the introduction and discussion section. For example, sentences between lines number 68-94 are poorly written and can be considered for deletion. Similarly, at various places in the manuscript, authors have failed to maintain consistency and coherence in the sentences. The acronyms used in the analysis need to be expanded when introduced in the manuscript—besides, the authors need to seriously check the in-out referencing. For example, WDI (2017) and Stock and Watson (2008) are not listed in the reference list. This article falls short on various grounds. | The manuscript has been reduced in size and introduction and discussion section has been made conscise.  Sentences between lines number 68-94 has been deleted.  All those acronyms used in the analysis has been expanded and reference list has also been updated |
| 1 | The methodology section of the manuscript is very confusing and needs serious attention. The authors have mentioned that both primary and secondary data sources have been carried out in the study. However, details on primary data source such as selection of households, sample adequacy, sampling design, statistical power are entirely missing from the analysis. The authors have selected 30 random samples from each country; however, in the study, N is equal to 700. The sample from each county is mentioned as 140. This creates massive confusion on the validity of the analysis. | The micro-level analysis has been completely removed friom the study. This study now only has macro-level analysis of 6 PICs, for which the data ranges from 1990-2019. This makes this section much more clear as suggested by reviewer 1.  Primary data is now not used,  Initially, this section benefitted from repeated sampling method available in STATA. Now primary data analysis is removed. |
| 2 | Who were the respondents in the survey? All women of reproductive age group 15-49 years or currently married women of the reproductive age groups. It would be suggested to consider rewriting the methods section in a more detailed manner. | The micro-level analysis(survey) has been completely removed from the study |
| 3 | The authors have explained a range of fertility theories in the literature review. However, in the analysis section, they have adopted indicators based on Davis and Blake (1956) and Ahlburg and Cassen (1993) to conduct analysis. Why so ? However, the later study finds no place in the literature review. | Fertility theories has been removed from literature review. Indicators suggested by Davis and Blake (1956) has been used in this study as it offered a quantitative framework to decompose fertility into its proximate determinants (social and economic variables). This framework is useful for performing comparative fertility analysis. Ahlburg and Cassen (1993) later draws work from Davis and Blake(1956). Both of these study has now been properly cited in the text |
| 4 | Another observation from the analysis is that the availability of knowledge of contraceptives by women is considered a proxy of the CPR for the micro-level data. Surprisingly, the authors did not ask whether the women were currently using contraceptives or not? Why so ? | The question was asked. However, the micro-level analysis(survey) has been completely removed from the study |
| 5 | The authors need to bring more clarity on the indicators chosen for the study. For example, what a reader should comprehend from the “Highest level of educational attainment of women”. Instead, in the table “Level of education” variable is mentioned. Are these two variables same ? Similarly, what is the meaning of “unmet need of pregnancy” ? does refer to failure in conceiving due to primary or secondary sterility or infertility, infecundity, or simply a mismatch between actual and desired fertility levels. Consider using the name for the indicators, which do not create confusion to the reader. | The indicators used for macro-level studies has now been clearly identified and explained. All the variables associated with micro analysis, as well as the micro level estimation has been removed from this study, which now removes ambiguity. |
| 6 | It is highly recommended to provide frequency distribution or descriptive statistics against each explanatory variable so that readers can comprehend sample adequacy for any empirical analysis. | Descriptive statistics against each explanatory variable, as well as the correlation matrix for macro variables has been now made available in the research |
| 7 | There are some shortcomings in describing the stepwise structure of the econometric models. What made the authors run these three OLS models on the survey data is absent in the study. The motivation of using different models should be underlined in the method section as to what hypothesis authors are attempting to testify through each model. | OLS models on the survey data has been removed. All the models used for macro analysis has been well described and justified, along with the key hypothesis being tested in the model |
| 8 | The variable “the number of kids to the women” depends on the age of the women. The fertility levels are dependent on the age at marriage and exposure to future fertility. How authors have accounted for the offset while dealing with OLS model. Also, considering the nature of the dependent variable, why do authors not employ the Poisson regression model on microanalysis? | The micro-level analysis(survey) has been completely removed from the study |
| 9 | The authors have employed the GMM estimation procedure assuming endogeneity in the model. To substantiate the premise, authors have cited study carried out by Arellano and Bond (1991). However, if such endogeneity persists or not in the case of PIC countries has not been empirically testified. I would suggest authors perform either the Hausman test or the Durbin-Wu-Hausman test to assess endogeneity in the present study's context. | GMM estimation has been removed. Fixed, random and Pooled OLS has been employed in this study. Appropriate testing such as Hausman test and Breusch Pagan Lagrangian multiplier has been employed to access the endogeniety in the study and for selection of the appropriate models |
| 10 | Since the study is based on several approximations and assumptions; therefore, authors must include a section on ‘Limitation of the study’. | Separate section on limitation of the study has been included in this paper |
| 11 | Lastly, the authors have failed to justify the study's title in the abstract, rationale and discussion section. | The title has been changed. Since this study has employed socio-economic and demohraphic variables, the title has been renamed to “Socio-Economic and Demographic determinants of Fertility in Six Selected Pacific Islands Countries: An Empirical Study”. The title has been also justified in abstract and discussion section |
| 12 | Considering grammatical errors and spelling mistakes in the manuscript, an immediate suggestion would be to get this manuscript copy-edited by some professional editor. | The entire article has been proof read again to check for any anomalies. |
